# Inclusion, universal design and universal design for learning in higher education: South Africa and the United States

**DOI:** 10.4102/ajod.v8i0.519

**Published:** 2019-07-29

**Authors:** Elizabeth M. Dalton, Marcia Lyner-Cleophas, Britt T. Ferguson, Judith McKenzie

**Affiliations:** 1 Department of Communicative Disorders, University of Rhode Island, Kingston, United States; 2 Dalton Education Services International, Hope Valley, Rhode Island, United States; 3 Disability Unit, Stellenbosch University, Cape Town, South Africa; 4 Special Education, National University, San Diego, United States; 5 Disability Studies Division, University of Cape Town, Cape Town, South Africa

**Keywords:** Universal design, universal design for learning, universal access, inclusion, inclusive education

## Abstract

Around the world, institutions of higher education are recognising their responsibilities to achieve the full inclusion of individuals with differing needs and/or disabilities. The frameworks of universal design (UD) and universal design for learning (UDL) offer unique ways to build inclusiveness in our systems. The role of UD and UDL to strengthen successful inclusion of persons with differing needs in higher education programmes is presented from literature, inclusive of national and international policies and resources. Examples from South African and US institutions of higher learning are shared. Discussions of online accessibility, environmental issues, professional development, barriers to inclusion and recommendations for future development in an international context provide a vision for developing inclusive learning environments in higher education.

## Background

Around the world, institutions of higher education are recognising their responsibilities to achieve the full inclusion of individuals with differing needs and/or disabilities. International treaties and conventions, such as the UN Convention on the Rights of Persons with Disabilities (2006) and, prior to that, the Universal Declaration of Human Rights (1948), have largely given impetus to the recognition of this inclusion of all people in society. The frameworks of universal access (UA), universal design (UD) and universal design for learning (UDL) offer unique ways to build inclusiveness especially in our educational systems.

Rethinking design for inclusiveness stems from pioneering ideas about design by Marc Harrison who, as a child, sustained traumatic brain injury. His experiences in interacting with the environment brought about this re-envisioning of physical space. He later became a professor of industrial engineering at Rhode Island School of Design and challenged the way design was created for ability and function according to the average person. ‘Universal design’ as a term came into use by Ronald Mace only in the 1970s. He also challenged average practices regarding design. The Center for Universal Design at North Carolina State University, which Mace helped establish, became the home of research around UD. Seven principles to guide UD were later identified (Burgstahler 2015). The seven principles of UD for designing products or services in the environment are as follows: equitable use; flexibility in use; simple and intuitive use; perceptible information; tolerance for error; low physical effort and size; and space for approach and use. By applying these principles, the use of products and services will be equitable for most people.

The concept of UDL stemmed originally from the UD principles, as well as from research in neuroscience on how the brain learns (Rose & Meyer 2002). Universal design for learning applies the concepts of accessibility and inclusion beyond physical environments, to design teaching and learning opportunities in ways that are varied, accessible and engaging for *all* students, including those with differing needs and/or disabilities. In this way, appealing to the broadest range of diversity in our student populations, the framework of UDL strives to remove discriminatory practices, as the learning needs of most students are taken into account when instruction is designed, thereby seeking to eliminate the need to ‘retrofit’ teaching practices with specialised accommodations. At the heart of UDL are its three core principles for instructional design: multiple means of engagement, multiple means of representation and multiple means of action and expression (Rose & Meyer 2002). The natural variation present within all classrooms is recognised and taken into account during the instructional design process and is periodically reviewed using UDL guidelines to check for efficacy of inclusive design (CAST 2018). Since the development of the UDL framework for instructional design by CAST, Inc., in the 1990s, UDL has been increasingly influential on educational systems and policies in the USA (ESSA 2015; HEOA 2008) and recently has been receiving attention internationally (Dalton 2018; Dalton & Lawrence 2010; Dalton, McPherson & Anderson 2011).

Since 1996, following investigation of discriminatory practices in South African (SA) education because of the apartheid system, a more inclusive system of education has been sought. Stereotyped attitudes, problems with accessibility and other challenges have made implementation of inclusive education quite elusive. South African professionals must engage with others in the field to learn different models and resources for implementing inclusion at all levels of education. To do so, knowledge and experience on methods and strategies to achieve inclusive education need to be sought. The experience and resources available in the USA in the areas of UD and UDL are significant, as the country of origin for these concepts. It is logical to build collaborative relationships between education professionals of the USA and SA to share challenges and develop solutions. Every two years, the International Association of Special Education (IASE) holds an international conference. Attracting hundreds of professionals from all corners of the globe and all levels of education, this forum shares research, information and resources to support students with diverse needs and disabilities around the world. This opinion paper is based upon the authors’ collaborative presentation at IASE 2017 in Perth, Australia, ‘Inclusion, Universal Design and Universal Design in Higher Education’. The intention is to present concepts and examples of UD and UDL and to discuss issues of barriers and potential solutions to help teachers, professors and others envision how they can take steps to reduce barriers to education in their own educational settings and build a system that is universally accessible and inclusive for *all*.

## Inclusive education policies and challenges

### Worldwide

Since the World Declaration on Education for All (UNESCO 1990) and the Salamanca Statement (UNESCO 1994), inclusive education has been a major focus worldwide. The Dakar Framework for Action (UNESCO 2000) and Policy Guidelines on Inclusion in Education (UNESCO 2009) added strength and urgency to this discussion. Specific core issues driving development of these actions include the (1) recognised need for access to education for all persons around the world, (2) recognised need for equity in educational rights and opportunities and (3) recognised right to receive adequate and appropriate accommodation and support for all students. With education’s movement towards providing general education for all students in the most ‘normalised’ environment, acquiring knowledge of inclusive learning and actually implementing inclusive education policies and strategies are critical for success. It is important to recognise, however, that educators’ and policymakers’ personal and professional understanding of inclusion around the world can vary greatly, depending upon where and who they are.

### United States

‘Inclusion’ is an educational term commonly used in the USA, primarily as the result of educational practices rather than policy. Inclusion, specifically, is not referenced in US laws governing general or special education (US Department of Education 1975, 2004). The *US Individuals with Disabilities Education Improvement Act of 2004* requires school districts to place students in the least restrictive environment (LRE) appropriate for their needs. In schools, general classroom settings are the *least restrictive* of all. Two federal civil rights laws, Section 504 of the *Rehabilitation Act of 1973* and the *Americans with Disabilities Act of 1990*, define equal rights and prohibit discrimination based on disability. In order to achieve equal rights in US education, the practice of inclusion is now widely supported throughout public education systems and beyond. In the USA, inclusive education is *understood* as having students of all varied needs and abilities educated together in general classroom settings (according to LRE guidelines), with the supports and services necessary for every student to receive educational benefit. This same understanding of inclusion may not, however, be common in other countries.

### South Africa

Inclusive education first appeared in SA education policy post-apartheid, after many years of race, colour and class inequalities. Schools were divided by race, disability and resources. Traditional conceptions of disability prevented children from attending school. The *Education White Paper 6: Special Needs Education. Building an Inclusive Education and Training System* (SA Department of Education 2001) introduced a new inclusive system of education recognising that learning needs may arise out of negative attitudes, stereotyping, inaccessible environments, inadequate policies and support services, and several other factors. This paper provided a broader framework that moved beyond the implementation, support and resource plans for inclusive education existing in SA. Fifteen years later, a study on teachers’ perceptions of the implementation of inclusive education in school systems in SA revealed clear challenges (Nel et al. 2016). Challenges cited include: (1) inadequate teacher training on inclusive education, (2) inefficient support in schools and (3) education department structures and the lack of community engagement. Clearly, while policies state the desire and need for inclusive education in SA, the realities of implementation make it an elusive goal.

In higher education, the need to put a framework in place for disability inclusion was recognised and was put in place in 2018. This framework is the first document of its kind based on disability support for students who have left the basic schooling system. The Strategic Disability Policy Framework in the Post-School Education and Training (PSET) System (Department of Higher Education and Training 2018) outlines three strategic objectives for the PSET sector. Firstly, striving to create a standardised enabling environment in the PSET sector to ensure systemic support based on the social model of disability is envisaged. Secondly, accessible teaching, learning, recreation and a supportive environment is envisioned. This framework acknowledges the need to foster UA and UD by removing barriers. Lastly, this framework strives to ensure coordination and cooperation across the various PSET systems.

## Potential solutions – Universal design and universal design for learning

In order to best address the growing need, interest and dedication to developing more inclusive learning environments across the educational spectrum, two key guiding concepts have been identified. Universal design and universal design for learning offer guidance in the development and maintenance of accessible physical and learning environments for *all* students.

Universal design’s foundation is based on seven principles for designing accessible environments: (1) equitable use, (2) flexibility in use, (3) simple and intuitive, (4) perceptible information, (5) tolerance for error, (6) low physical effort and (7) size and space for approach and use (Center for Universal Design 1997). Additional UD information is available at https://projects.ncsu.edu/ncsu/design/cud/.

Universal design for learning is a curriculum and instructional design framework based in neuroscientific research and focused on how the brain recognises, processes, organises, evaluates and responds to varied types of information (Meyer, Rose & Gordon 2014). Its three core principles, specifically *multiple means of representation, multiple means of action and expression* and *multiple means of engagement*, are enhanced and clarified by the UDL guidelines (Hall, Strangman & Meyer 2003). While UDL was first developed primarily to address instructional design in K–12 education, most recently CAST and the UDL Implementation and Research Network have focused on the challenges of equity and inclusion at higher education levels. Additional information and materials on UDL guidelines, research, resources and UDL in higher education are available at http://www.cast.org/.

Together, the principles and guidelines for implementing UD and UDL provide practical tools to aid professionals in designing universally accessible classroom and online environments wherever educators seek to expand and implement inclusive instructional systems.

## Universal access, inclusion and higher education

The SA *National Plan for Higher Education* (SA Department of Education 2001) encouraged the increased intake of students with disabilities and its *White Paper on Post-School Education and Training* (2013) focused attention on the PSET sector. Despite these efforts, effective inclusion in higher education for those with disabilities has been inconsistent. While disability supports for physical issues (i.e. as text conversion, Braille, sign language, etc.) exist in most SA higher education institutions and in some technical vocational education and training colleges, difficulties regarding disclosure based on psychological and ‘hidden’ factors (De Cesarei 2015) are prevalent. It is therefore important to develop a more universal approach to disability support systems in higher education, in part as a result of lingering effects of inequalities built during apartheid, as well as the inherent natural diversity of disabilities overall. Some universities are moving towards UA policies focused on function and not disability by applying the principles of UD and UDL (Burgstahler 2015; Center for Universal Design 1997; Dalton, McKenzie & Kahonde 2012; Howell 2005, 2015). Digital access and online learning platforms may, however, exclude those with disabilities because of adaptive device costs, extensive support needs and inaccessible Internet design (Perez, Grant & Dalton 2016; Watling 2011). In order to ensure equity of access in higher education, universities and other post-secondary institutions must consider physical and programmatic access, content readability, personal usability and appropriate individual and system-based supports in order to achieve the goal of inclusive education.

## Higher education programmes – Four examples of challenges and solutions

### University of Cape Town, South Africa

While the University of Cape Town (UCT) has an active and responsive disability service, the challenge of equitable access to the online learning environment remains. The technology that holds so much promise for increased accessibility contains within it the possibility of further exclusion of students who access text in different ways, especially those with visual impairment (Schmetzke 2001). In the UCT postgraduate diploma programme in Disability Studies, students with visual impairments faced specific accessibility challenges, especially in relation to learning online. These included the need for: (1) print resources to be accessible and on time, (2) appropriate assistive technology software to support access to online materials, (3) tests and quizzes to be accessible in a timely manner and (4) the lack of adequate home Internet connections to support access. While significant steps were taken to mitigate these barriers, academic staff believe that such issues could have been avoided if UDL had been used in designing a learning programme with *all* students in mind. Moreover, changes that would improve online accessibility would have positive effects for students beyond those with visual impairment in providing access to the curriculum (Howell, McKenzie & Chataika 2018). What is needed is a systemic change at university level rather than within specific programmes. This is now starting to happen as library, information technology and disability services as well as academic programmes are collaborating to address online accessibility within a UDL framework.

### Stellenbosch University, South Africa

Research on the challenges of students with differing needs and/or disabilities in higher education settings outside of the USA is relatively rare. A study of students’ experiences of inclusion and exclusion in higher education at Stellenbosch University (SU) revealed both challenges and strengths in the disability support system (Lyner-Cleophas 2016). Challenges at SU include: (1) insufficient planning for inclusion from the start from a disability perspective, (2) the need for disability to be viewed as part of the transformation occurring in SA society, (3) faculty and staff are not always disability aware, (4) existing subtle disability exclusion, as disability may be viewed as a disability office matter only and (5) some people think UD and UA are ideal and too expensive (Lyner-Cleophas 2016). Strengths identified include: (1) some staff had knowledge of UA design and its advantages over retrofitting, (2) access to some assistive technology is available through the SU disability unit, (3) the disability unit support team actively engages students and staff when difficulties occur and (4) inclusion access is as good for staff as for students (Lyner-Cleophas 2016). Efforts continue by disability support personnel to provide awareness training and supports to broaden UA implementation at SU.

Recently, a new disability access policy was developed at SU (Stellenbosch University 2018). This policy is not for students alone but applicable to students, staff and visitors to campus. Universal design elements are considered as well as the notion of UA. The principles of UD are incorporated at policy level and applicable to the teaching and learning environments. These principles are the same for those indicated as UD principles at the start. This also involves reasonable accommodation and the practicality of what is possible given physical and financial constraints in SA reality. Designing for all (and not people with disabilities only) is an idea that is setting in, as this is cost-effective in the long run and engages the diversity of people in more ways than just race and language. Stellenbosch University is a campus in town and closely engages with the Stellenbosch Municipality with reference to access in physical spaces such as pavements and parking, which are mainly municipal competencies. The municipality has also drafted a UA policy in line with UA principles as it strives towards the broader inclusivity of people (Stellenbosch Municipality 2015). Incorporating good practices starts with the acknowledgement of what is good for most people as well as instituting good policy frameworks. A value added to the Stellenbosch University Vision 2040 is the well-being of its staff and students. To this end, SU strives towards creating an environment that is accessible to the broadest range of students, staff and visitors to campus.

### National University, United States

At National University (NU), educator training programmes are primarily or partially online and must integrate California Standards for the Teaching Profession and Teaching Performance Expectations (TPEs). Recently revised TPEs reference and address the concepts of UDL. National University’s Teacher Education (TED) and Special Education (SPED) programmes are working together to include these UDL concepts in their curricula. Faculty from TED are learning about UDL and are anxious to infuse UDL core principles through co-planning with SPED. The nature and depth of UDL will need to be thoroughly discussed and internalised by faculty, as it is essential that agreement is reached on what the acquisition of UDL knowledge and skills will involve and how best to prepare NU’s teacher candidates in these principles. Identification of exemplary practices in UD and UDL, especially for inclusion of students with severe disabilities, is needed. Ongoing, in-depth discussion of UDL and the UD principles by faculty will ensure both learning and application of these principles by novice teachers. Students who have identified disabilities, and who qualify, may be afforded additional accommodations to support their success. Candidates’ needs are addressed by Student Accessibility Services and may include note takers, extra time on examinations and interpreters for the deaf. All online materials are compliant with federal law regarding accessibility and therefore can be viewed and/or heard.

### University of Rhode Island, United States

Blended learning, through both online and face-to-face instruction, is growing in US higher education, and along with it come the challenges of establishing and sustaining equity and accessibility in online environments. At University of Rhode Island (URI), the online learning system, Sakai, integrates many features to improve the accessibility of online materials. Features include ‘how to make images more accessible’, ‘how to make videos and audio files more accessible’, ‘how to make links accessible’, ‘use of background and text colour’, ‘how to structure a document for accessibility’ and others.

The UDL framework is used to address the diversity of student learning needs. In one example, the framework of UDL is applied in preparing speech language pathology graduate students through their course in Augmentative and Alternative Communication (AAC). This blended learning experience, inspired by UDL principles, is hosted through the open-source Sakai learning management system (LMS). It uses multimedia resources, open-source materials, online learning tools and face-to-face classes to offer students multiple means of content representation and multiple means for demonstration of content competence through project-based learning and various online discussion tools. Online reflection journals demonstrate students’ engagement with course content and with assignments using varied materials and assessments. Students evaluated overall course satisfaction as very high. All students achieved high levels of academic performance in the course, as well.

Across the USA, institutions are recognising that inclusion and equity of access are a priority, and these institutions continue to need support in achieving greater accessibility. CAST developed the website *UDL on Campus* to provide connections, guidelines and resources for higher education. A rich collection of information is available at http://www.udloncampus.cast.org.

## Ethical considerations

This article followed all ethical standards for carrying out research without direct contact with human or animal subjects.

## Discussion

The inclusion of students with disabilities in the mainstream of education, together with their non-disabled peers, has been clearly shown to be preferred policy in both the USA and in SA, as evidenced by the wealth of policy statements and legislation in both countries, as well as in worldwide policies and educational equity-related guidelines (*Americans with Disabilities Act* 1990; Department of Higher Education and Training [SA] 2018; ESSA 2015; HEOA 2008; SA Department of Education 2001; UNESCO 2000, 2009). While such policies, laws and guidelines have existed in both the USA and in SA for at least 15 years or more, the degree of implementation within and between these countries varies greatly. Some of the variation may likely be because of the differing histories of the two countries, the strong influence of apartheid in SA for so many years, and differences in development and implementation of federal guidance for inclusion. In the USA, the challenges of racial, ethnic and disability-related discrimination continue to emerge and impact the educational systems, even with more than 40 years having passed since the passage of the *Rehab Act of 1973*, which first required equal access to education facilities and programmes for students with disabilities. In SA, while *Education White Paper 6* (2001) provided a new vision for the inclusion of students with disabilities in mainstream education, it was not until 2018, with passage of the Strategic Disability Policy Framework in the PSET System, that inclusive educational policies were articulated for higher education. While both countries continue to face challenges to the achievement of equity for all, the programmatic examples shared here from four different higher education institutions bear both similarities and areas of significant difference.

The US higher education institutions cited both have robust LMSs that support broad online instruction systems. These systems are enabled with accessibility features and guidelines that can be activated in order to present materials and instruction in an accessible format. The SA universities are not widely using such systems yet and are challenged to make individual adjustments and accommodations for each student in need. There will always be some level of need for providing customised modifications and accommodations for students with complex and/or unique learning challenges; however implementation of systems that have been designed to offer options for variation and accommodation for both teachers and students can greatly reduce the barriers faced by students with disabilities in higher education. Use of systems that integrate accessibility options is very much in line with the concepts and principles of UDL.

There is emerging research-based evidence that UD and UDL can positively influence the level and experience of learning for students at various levels of education (Black et al. 2015; Burgstahler 2015; Katz 2013). Literature also reveals some scepticism about the sustainability of impact of UDL on the field (Edyburn 2010). At the institutions in the USA and SA referenced earlier, it is clear that the both UD and UDL are being embraced to help guide to some extent the development of more inclusive learning environments for all students. Through the use of technology at UCT, individuals with visual impairments can access and participate in professional development programmes that would otherwise have been inaccessible. In response to research conducted at SU, the campus environment is increasingly aware of and working to remove the physical and instructional barriers existing for students with disabilities, embracing the ideas of ‘designing for all’. At NU, faculty development integrates instruction and support to bring UDL integration into the curriculum, and the challenge of addressing UD and UDL in state and national standards is receiving great attention. Through the embedded accessibility features of the Sakai LMS and using a blended learning model to maximise options for multiple means of representation of content, engagement in learning and expression of knowledge through varied means, students in the AAC course at the URI use multimedia, face-to-face discussion, online reflection with peers and project-based learning to complete course requirements, which are designed through a UDL-inspired lens.

As more and more institutions of higher learning take to heart their responsibilities to offer inclusive, equitable and non-discriminatory learning opportunities for all students, they are finding that the frameworks of UD and UDL provide helpful guidance for the design of physical environments and instructional opportunities that are accessible and engaging to a broad range of learners from the start. Resources such as the CAST (http://www.cast.org), Universal Design for Learning Implementation and Research Network (UDL-IRN) (http://www.udl-irn.org), the Inclusive Learning Network of International Society for Technology in Education (ISTE) (http://www.facebook.com/ISTEInclusiveLearning/), the UDL Special Interest Group of SITE (http://www.facebook.com/groups/SITEUDLSIG/) and the National Center for Accessible Educational Materials (http://www.aem.cast.org/) offer a wealth of information, publications and professional learning opportunities to expand professional understanding and integration of UDL.

## Recommendations

Based on their individual and shared professional work, the authors offer the following recommendations for higher education:

- Focus on the functional needs of students, staff and campus visitors and do not judge based upon labels used. Students vary greatly in the nature of their needs, even within a particular area of disability.
- Make inclusion and accessibility a campus-wide dialogue. Everyone needs to be included in identifying the needs and the solutions. It is not an endeavour for the disability units or teaching staff only.
- Build a systemic foundation using inclusive models for educational design, such as UD and UDL, applicable to facilities management, teaching faculty, support services and admission procedures.
- Leverage technology to support inclusion, rather than letting it become a barrier.
- Reach out to others for ideas and help in addressing challenges. There are many great resources and organisations that support inclusive education principles, and we recommend that higher education institutions use them.

### Note to professionals

In 2019, the 16th Biennial IASE conference took place at Sebastian Kolowa Memorial University in Magamba, Tanzania, East Africa, from 13 to 17 July 2019. The theme was ‘Empowering Persons with Disabilities: Developing Resilience and Inclusive Sustainable Development’. Information about IASE, membership and biennial conference registration is available at http://www.iase.org/.

## Conclusion

The challenges to achieving comprehensive inclusion in higher education for students with diverse needs and disabilities are significant; however, tools, strategies, examples and guidelines exist that can lead to success, if applied creatively and effectively. The four university examples, based on experience, highlight some of the challenges and potential solutions. Physical and programmatic inaccessibility, lack of timeliness, equipment mismatches and excessive costs can keep students from being adequately supported in their studies. Lack of awareness, misunderstandings, lack of knowledge and training, and lack of resources are some of the reasons why higher education institutions and faculty are not sufficiently or appropriately supportive of inclusion. However, models for success in designing and implementing inclusive educational systems in higher education are emerging. New digital resources can be leveraged, and diversity can be celebrated rather than feared. Faculties of teacher preparation and professional service preparation programmes around the world must embrace the idea that *all* upcoming teachers need to recognise, understand and embrace inclusive education practices. Sharing professional experiences and practical ideas for implementation is a good place to begin.
